# COVID‐19 Vaccine Effectiveness Against Medically Attended Symptomatic SARS‐CoV‐2 Infection Among Target Groups in Europe, October 2024–January 2025, VEBIS Primary Care Network

**DOI:** 10.1111/irv.70120

**Published:** 2025-05-21

**Authors:** Charlotte Laniece Delaunay, Nuno Verdasca, Susana Monge, Lisa Domegan, Noémie Sève, Silke Buda, Adam Meijer, Héloïse Lucaccioni, Miriam López Torrijos, Adele McKenna, Vincent Enouf, Ralf Dürrwald, Eline In't Velt, Mª Ángel de Valcárcel Laiglesia, Charlene Bennett, Shirley Masse, Annika Erdwiens, Mariette Hooiveld, Ivan Mlinarić, Gergő Túri, Ana Paula Rodrigues, Iván Martínez‐Baz, Mihaela Lazar, Neus Latorre‐Margalef, Vitor Borges, Marlena Kaczmarek, Sabrina Bacci, Esther Kissling, European primary care VE group

**Affiliations:** ^1^ Epiconcept Paris France; ^2^ Infectious Disease Department National Institute of Health Dr. Ricardo Jorge Lisbon Portugal; ^3^ National Centre of Epidemiology Institute of Health Carlos III Madrid Spain; ^4^ Health Service Executive Health Protection Surveillance Centre Dublin Ireland; ^5^ Sorbonne Université, INSERM, Institut Pierre Louis d'épidémiologie et de Santé Publique (IPLESP) Paris France; ^6^ Department of Infectious Disease Epidemiology, Respiratory Infections Unit Robert Koch Institute Berlin Germany; ^7^ National Institute for Public Health and the Environment Centre for Infectious Disease Control Bilthoven the Netherlands; ^8^ Subdirección General de Epidemiología y Vigilancia de la Salud Valencia Spain; ^9^ Centre National de Référence Virus des Infections Respiratoire (CNR VIR), M3P Unit, Institut Pasteur Université Paris Cité Paris France; ^10^ National Reference Centre for Influenza Robert Koch Institute Berlin Germany; ^11^ Department of Epidemiology Regional Health Council, IMIB Murcia Spain; ^12^ National Virus Reference Laboratory University College Dublin Ireland; ^13^ Unite des Virus Emergents (UVE: Aix‐Marseille Univ, Universita di Corsica, IRD 190, Inserm 1207, IRBA) Marseille France; ^14^ Nivel Utrecht the Netherlands; ^15^ Croatian Institute of Public Health Zagreb Croatia; ^16^ National Laboratory for Health Security, Epidemiology and Surveillance Centre Semmelweis University Budapest Hungary; ^17^ Epidemiology Department National Institute of Health Dr. Ricardo Jorge Lisbon Portugal; ^18^ Instituto de Salud Pública de Navarra – IdiSNA – CIBERESP Pamplona Spain; ^19^ Cantacuzino National Military Medical Institute for Research and Development Bucharest Romania; ^20^ Department of Microbiology Public Health Agency of Sweden Stockholm Sweden; ^21^ European Centre for Disease Control and Prevention Stockholm Sweden

**Keywords:** COVID‐19, Europe, SARS‐CoV‐2, vaccine effectiveness

## Abstract

We estimated the effectiveness of 2024/25 COVID‐19 vaccination against medically attended SARS‐CoV‐2 infection in Europe, among target groups. We included 3204 patients (8/139 cases vaccinated: 6%; 517/3065 controls vaccinated: 17%) from a multicentre, test‐negative design study at primary care level. Vaccine effectiveness was 66% (95% CI: 34–85) overall, 73% (95% CI: 21–94) and 54% (95% CI: −3 to 83) in the first and second months post‐vaccination, respectively. Overall vaccine effectiveness was 67% (95% CI: 33–86) among older adults (≥ 60 or ≥ 65 years). This relatively high COVID‐19 VE (compared with previous seasons), as well as trends by time since vaccination, should be confirmed with additional data, as sample size was low.

## 1 Introduction

In Europe, the wave of SARS‐CoV‐2 infections observed in the summer of 2024 was followed by decreased activity in the autumn/early winter [1]. Many European countries launched COVID‐19 vaccination campaigns between September and December 2024, recommending primarily newly authorised vaccines adapted to SARS‐CoV‐2 lineages JN.1 or KP.2 (Comirnaty JN.1, Comirnaty KP.2, Nuvaxovid JN.1 and Spikevax JN.1) [2]. These country‐specific campaigns targeted, for example, older adults, people with chronic conditions, pregnant women and certain professional groups (Table S1).

We estimated interim 2024/25 season COVID‐19 vaccine effectiveness (VE) against medically attended, PCR‐confirmed SARS‐CoV‐2 infection in Europe among target groups, using primary care data from the Vaccine Effectiveness, Burden and Impact Studies (VEBIS) [3].

## 2 Methods

VEBIS Primary Care is a test‐negative design, case–control study of COVID‐19 VE conducted in 11 European study sites (Table S1). The methods have been described elsewhere [3, 4, 5]. Briefly, the study population comprises patients consulting primary care physicians for acute respiratory infection [6] who are tested for SARS‐CoV‐2 using RT‐PCR within 10 days of symptom onset. Cases are symptomatic patients testing positive, and controls those testing negative. Oro‐/nasopharyngeal samples are also tested for other respiratory pathogens and physicians collect demographic, clinical and vaccination information via interviews and/or linkage to electronic medical records and vaccination registries. All or a random/systematic sample of SARS‐CoV‐2‐positive samples are selected for genetic sequencing. Sequences are uploaded to GISAID [7] and phylogenetic and amino acid substitution analyses are performed centrally at the Instituto Nacional de Saúde Doutor Ricardo Jorge, Lisbon, Portugal.

We included patients whose symptom onset occurred ≥ 14 days after the start of the autumn/winter 2024/25 COVID‐19 vaccination campaign in their country (Table S1) and before 19 January 2025. We excluded controls with symptom onset prior to the week of symptom onset for the first COVID‐19 case in their study site.

We selected patients who were part of clinically vulnerable target groups for COVID‐19 vaccination according to country‐specific recommendations (Table S1). We excluded children ≥ 6 months and < 5 years of age because the number of recommended COVID‐19 vaccine doses differed from other age groups [8]. We further applied regular study exclusion criteria (Table S2), including the exclusion of study sites with ≤ 10 cases or controls.

We defined as vaccinated patients who received any dose of COVID‐19 vaccine ≥ 14 days before symptom onset and after the start of the vaccination campaign in their study site. Unvaccinated patients were not vaccinated during the campaign or in the 6 months before. People vaccinated on the day of symptom onset were classified as unvaccinated.

To minimise small sample size bias, we used Firth's penalised logistic regression [9] to estimate COVID‐19 VE as 1 minus the adjusted odds ratio of vaccination among cases and controls. We estimated VE among the whole target group for vaccination and among older adults comprising their country's recommended age‐specific target group, i.e., aged ≥ 60 or ≥ 65 years depending on the country (Table S1). We estimated VE overall and by time since vaccination (TSV) using 30‐day (14–29; 30–59), 42‐day (14–41; 42–83) and 60‐day (14–59) intervals, as sample size allowed. Varying the intervals minimises the sensitivity of our results to TSV categorisation and allows comparison with various studies.

We adjusted for the following a priori confounders and explored all possible combinations of the following functional forms of variables: study site (categorical variable), symptom onset date (categorical variable; restricted cubic spline with 3–5 knots), age (continuous variable; categorical variable with different age bands; restricted cubic spline with 3–5 knots) and sex (binary variable). We performed complete case analyses.

For each analysis, we chose the best‐fitting, fully adjusted model, using the Akaike information criterion. We also examined odds ratios and their standard errors for unstable results.

In the sensitivity analyses, we explored whether the presence of chronic conditions (at least one of diabetes, immunodeficiency, lung disease or heart disease) could confound our VE estimates by excluding patients with missing information on this variable and estimating VE adjusted and unadjusted for chronic conditions. We also replicated all VE analyses using a cut‐off of vaccination 7 days prior to symptom onset (rather than 14 days) for considering someone as being immunised. We excluded influenza‐positive controls to correct for potential confounding bias due to correlated COVID‐19 and influenza vaccination behaviours [10]. We excluded from TSV analyses vaccinated patients whose 2024/25 season vaccination date was imputed (as, in one study site, imprecise vaccination dates were imputed as the last day of the month of vaccination). Finally, we replicated all analyses using standard logistic regression.

For each analysis, we did not estimate VE if either: the number of sites was higher than the number of cases (or controls) divided by 10, or if there were < 20 vaccinated or unvaccinated patients. We also assumed small sample size bias if the VE estimates from standard logistic regression differed by ≥ 10% from those obtained with penalised logistic regression and did not show these estimates.

## 3 Results

After restrictions (Table S2), we included 3204 patients comprising 139 (4%) cases and 3065 (96%) controls from five study sites, with onset dates spanning from 1 October 2024 to 9 January 2025. The median age was 66 years (interquartile range [IQR]: 53–73) among cases and 63 years (IQR: 47–72) among controls (Table S3). Sixty percent (81/134) of cases and 70% (2099/3019) of controls had at least one chronic condition.

Among cases, 6% (8/139) received COVID‐19 vaccination this autumn/winter of 2024/2025, compared with 17% (517/3065) among controls (Table S3). The median number of days since last vaccination among the vaccinated was 42 days (IQR: 34–47) among cases and 41 days (IQR: 27–55) among controls. Among vaccinated patients with vaccine brand information, 83% (5/6) of cases and 97% (469/486) of controls received Comirnaty vaccines, and among those, 80% (4/5) of cases and 60% (283/469) received Comirnaty JN.1 where vaccine antigen was known. As of January 2025, 22% (31/139) of SARS‐CoV‐2‐positive samples were sequenced. Ninety‐four percent (29/31) belonged to the BA.2.86 lineage, of which 66% (19/29) belonged to the sub‐lineage XEC and 24% (7/29) to KP.3.

Overall COVID‐19 VE was 66% (95% CI: 34%–85%) among the whole target group for vaccination (Table 1). The VE was 73% (95% CI: 21%–94%) 14–29 days post‐vaccination and 54% (95% CI: −3% to 83%) 30–59 days post‐vaccination. Among older adults, COVID‐19 VE was 67% (95% CI: 33%–86%) overall, 68% (95% CI: 3%–93%) 14–29 days post‐vaccination and 59% (95% CI: 0%–87%) 30–59 days post‐vaccination. Estimates for 42‐day and 60‐day intervals are presented in Table 1.

**TABLE 1:** Pooled vaccine effectiveness against medically attended, symptomatic, PCR‐confirmed SARS‐CoV‐2 infection among target groups for vaccination, *VEBIS primary care study*, Europe, October 2024–January 2025.

Population group and TSV analysis	TSV (in days)	Cases	Median TSV (in days) among cases (IQR)	Controls	Median TSV (in days) among controls (IQR)	VE (95% CI)
Whole target group	Unvaccinated	131	—	2548	—	—
	Any	8	42 (34–47)	517	41 (27–55)	66 (34–85)
30‐day TSV intervals	14–29	2	18 (17–20)	159	22 (18–26)	73 (21–94)
	30–59	5	43 (40–46)	255	44 (36–51)	54 (−3–83)
42‐day TSV intervals	14–41	4	30 (20–39)	264	27 (21–33)	70 (30–90)
	42–83	4	48 (45–56)	249	55 (48–65)	53 (−13–85)
60‐day TSV interval	14–59	7	40 (30–44)	414	34 (24–46)	64 (28–85)
Older adults (part of the age‐specific target group for vaccination)	Unvaccinated	79	—	1306	—	—
	Any	7	40 (30–49)	448	41 (27–56)	67 (33–86)
30‐day TSV intervals	14–29	2	19 (17–20)	131	22 (18–26)	68 (3–93)
	30–59	4	43 (40–47)	225	44 (36–51)	59 (0–87)
42‐day TSV intervals	14–41	4	30 (20–39)	225	27 (21–33)	66 (18–89)
	42–83	3	51 (49–62)	219	55 (49–65)	64 (4–90)
60‐day TSV interval	14–59	6	40 (26–45)	356	35 (25–46)	65 (25–86)

Abbreviations: CI, confidence interval; IQR, interquartile range; TSV, time since vaccination; VE, vaccine effectiveness; VEBIS, Vaccine Effectiveness, Burden and Impact Studies.

The majority of sensitivity analyses resulted in absolute changes in VE point estimates of ≤ 3% (Table S4a–e). Using 7 days post‐vaccination to define someone as immunised increased VE by 6%–13% for any TSV and in early TSV intervals and decreased VE by 2%–3% in later intervals. When excluding patients with imputed vaccination dates, who represented 2% (10/517) of the vaccinated, absolute changes in VE by TSV were ≤ 9%.

## 4 Discussion

COVID‐19 vaccines administered during the autumn/winter 2024/25 vaccination campaigns in Europe showed approximately 65% effectiveness against symptomatic SARS‐CoV‐2 infection in primary care among target groups, in the 3 months post‐vaccination, although confidence limits overlapped. VE declined from 73% in the first month to 54% in the second month. Estimates were similar (67% overall) among older adults, as they contributed substantially to VE among the whole target population (representing 87% of vaccinated patients).

Sample size and precision were low for these analyses, presumably due to reduced SARS‐CoV‐2 circulation in the autumn and early winter, and to the relatively low proportion of patients vaccinated against COVID‐19 in the 2024/25 season. Therefore, caution is warranted when interpreting these estimates. Nevertheless, all estimates presented here fulfil criteria established a priori to minimise small sample size bias. High SARS‐CoV‐2 circulation in the summer could have led to lower vaccine uptake and lower risk of infection among unvaccinated patients with prior infection, thereby biasing VE downwards [11]. Given the high VE, this potential bias likely had little impact on our results.

We present timely, post‐marketing, independent estimates of seasonal COVID‐19 VE against symptomatic infection at European level. Our findings suggest a higher VE than in the previous season (VE was 40% [95% CI: 26%–53%] for the same period and population in 2023/24 [4]). This may be due to a better match of the vaccine to circulating viruses in 2024/25 compared with 2023/24 [12]. In this season, Omicron XBB.1.5 vaccines were predominantly used in our study, whereas among sequenced samples of cases nearly 40% of viruses belonged to Omicron BA.2.86 and descendants. Indeed, the 2023/24 XBB‐lineage‐specific VE point estimate among older adults in the target group for vaccination within 6 weeks post‐vaccination was higher at 63% (95% CI: −2 to 90), although precision was low.

The antigen composition of 2024/25 COVID‐19 vaccines varied slightly between the United States, where the Food and Drug Administration recommended KP.2‐adapted vaccines [13], and Europe (both JN.1‐ and KP.2‐adapted vaccines recommended, with JN.1 vaccines authorised earlier [2]). Limited serological evidence suggests that JN.1 vaccines may perform better against SARS‐CoV‐2 lineages circulating this season (e.g., KP.3) [14], and our VE results are slightly higher than early US estimates of the Comirnaty KP.2 VE against outpatient visits [15] and higher than VE against urgent care and emergency care visits (noting these can include hospitalisation) [16] in the United States.

These results are to be confirmed later in the 2024/25 season, with additional data, as sample size was low, and longer TSV intervals. Other research questions of interest include the estimation of VE against specific lineages circulating this season and by vaccine antigen component.

## Author Contributions

**Charlotte Laniece Delaunay:** conceptualisation, investigation, writing – original draft, methodology, validation, visualisation, writing – review and editing, formal analysis, project administration, data curation. **Nuno Verdasca:** investigation, writing – review and editing, methodology, formal analysis, data curation. **Susana Monge:** investigation, writing – review and editing, data curation, project administration. **Lisa Domegan:** investigation, writing – review and editing, data curation, project administration. **Noémie Sève:** investigation, writing – review and editing, data curation, project administration. **Silke Buda:** investigation, writing – review and editing, data curation, project administration. **Adam Meijer:** investigation, writing – review and editing, data curation, project administration. **Héloïse Lucaccioni:** investigation, methodology, validation, writing – review and editing, project administration, data curation. **Miriam López Torrijos:** investigation, writing – review and editing, data curation. **Adele McKenna:** investigation, writing – review and editing, data curation, project administration. **Vincent Enouf:** investigation, writing – review and editing, data curation, project administration. **Ralf Dürrwald:** investigation, writing – review and editing, project administration, data curation. **Eline In't Velt:** investigation, writing – review and editing, project administration, data curation. **Mª Ángel de Valcárcel Laiglesia:** investigation, writing – review and editing, data curation. **Charlene Bennett:** investigation, writing – review and editing, data curation. **Shirley Masse:** investigation, writing – review and editing, data curation. **Annika Erdwiens:** investigation, writing – review and editing, project administration, data curation. **Mariëtte Hooiveld:** investigation, writing – review and editing, project administration, data curation. **Ivan Mlinarić:** investigation, writing – review and editing, project administration, data curation. **Gergő Túri:** investigation, writing – review and editing, project administration, data curation. **Ana Paula Rodrigues:** investigation, writing – review and editing, project administration, data curation. **Iván Martínez‐Baz:** investigation, writing – review and editing, project administration, data curation. **Mihaela Lazar:** investigation, writing – review and editing, project administration, data curation. **Neus Latorre‐Margalef:** investigation, writing – review and editing, project administration, data curation. **Vitor Borges:** investigation, writing – review and editing, data curation, formal analysis. **Marlena Kaczmarek:** conceptualisation, funding acquisition, writing – review and editing, project administration, supervision, resources. **Sabrina Bacci:** conceptualisation, funding acquisition, writing – review and editing, supervision, resources, project administration. **Esther Kissling:** conceptualisation, investigation, funding acquisition, methodology, validation, writing – review and editing, project administration, data curation, supervision, resources. **The VEBIS Primary Care Vaccine Effectiveness Group:** conceptualisation, investigation, funding acquisition, methodology, validation, writing – review and editing, project administration, data curation, resources.

## Ethics Statement

Official ethical approval was not required in Spain (national), Ireland or the Netherlands, as this study was classified as routine care/surveillance. Other study sites received local ethical approval from a national or regional review board: Croatia: (class 030‐02/23‐01/1); France: Ethics statement 471393; Germany: EA2/126/11; Hungary: IV/1885‐5/2021/EKU; Navarre region: (Spain): Navarra, Spain: approved by the Ethical Committee for Clinical Research of Navarre (PI2024/150); Portugal: no registration number given; Romania: ce199/2022; Sweden: 2006/1040‐31/2 revised Drn2024‐06069‐02.

## Consent

Patient consent was not required in Ireland or Spain. Verbal consent was required for all other study sites, with the exception of Germany, Hungary and Romania, where written consent was required.

## Conflicts of Interest

The authors declare no conflicts of interest.

## Supporting information


**Table S1.** Autumn/winter 2024/25 COVID‐19 vaccination campaign start date and target group by study site, *VEBIS primary care study*, Europe, September 2024–January 2025.

**Table S2.** Study eligibility flowchart, *VEBIS primary care study*, Europe, October 2024–January 2025.

**Table S3.** Baseline characteristics of patients included in the *VEBIS primary care study*, Europe, October 2024–January 2025.

**Table S4a.** Sensitivity analyses of pooled vaccine effectiveness against medically attended, symptomatic, PCR‐confirmed SARS‐CoV‐2 infection among target groups for vaccination, *VEBIS primary care study*, Europe, October 2024–January 2025.
**Table S4b.** Sensitivity analysis: Excluding patients missing information on chronic condition, with and without adjustment.
**Table S4c.** Sensitivity analysis: Using 7 days post‐vaccination to consider someone as vaccinated.
**Table S4d.** Sensitivity analysis: Excluding influenza positive controls.
**Table S4e.** Sensitivity analysis: Excluding vaccinated patients with an imputed vaccination date from TSV analyses.

## Data Availability

The data that support the findings of this study are available from the corresponding author upon reasonable request.
