# Supplementary material for: COVID‐19 Vaccine Effectiveness Against Medically Attended Symptomatic SARS‐CoV‐2 Infection Among Target Groups in Europe, October 2024–January 2025, VEBIS Primary Care Network
Source: Influenza Other Respir Viruses. 2025 May 21;19(5):e70120. doi: 10.1111/irv.70120 (PMC12093050; doi:10.1111/irv.70120)
Supplement: Supplementary file 1 — Table S1. Autumn/winter 2024/25 COVID‐19 vaccination campaign start date and target group by study site, VEBIS primary care study, Europe, September 2024–January 2025. [file IRV-19-e70120-s003.docx]

**Table S1. Autumn/winter 2024/25 COVID-19 vaccination campaign start date and target group by study site, *VEBIS primary care study*, Europe, September 2024–January 2025**

| Study site | Start of autumn/winter 2024/25 COVID-19 vaccination campaign | Clinically vulnerable target groups for COVID-19 vaccination (1) |
| --- | --- | --- |
| Croatia (2) | October 21, 2024 | - Any age and chronic condition  - Pregnant women  - Age ≥65 |
| France | October 15, 2024 | - Any age and chronic condition  - Pregnant women  - Age ≥65 |
| Germany | October 1, 2024 | - Any age and chronic condition  - Age ≥60 |
| Hungary | December 2, 2024 | - Any age and chronic condition  - Pregnant women  - Age ≥60 |
| Ireland | September 30, 2024 | - Any age and chronic condition  - Pregnant women  - Age ≥60 |
| The Netherlands | September 16, 2024 | - Any age and chronic condition  - Age ≥60 |
| Portugal | September 20, 2024 | - Any age and immunodeficiency  - Age ≥5 and chronic condition  - Pregnant women  - Age ≥60 |
| Spain, Navarre region | October 14, 2024 | - Any age and chronic condition  - Pregnant women  - Age ≥60 |
| Spain, national | Between September 23, 2024 and October 23, 2024 (variations by region and age group) | - Any age and chronic condition  - Pregnant women  - Age ≥60 |
| Romania | *No seasonal COVID-19 vaccination campaign (excluded from analyses)* | - |
| Sweden | October 15, 2024 | - Age ≥18 and chronic condition  - Pregnant women  - Age ≥65 |
| Abbreviation: VEBIS, Vaccine Effectiveness, Burden and Impact Studies.  (1) The list of chronic conditions used to define the target group varied across study sites.  (2) There was no defined vaccination campaign in Croatia, as COVID-19 vaccination occurred year-round.  We used the date of announcement of the updated autumn/winter 2024/25 vaccination recommendations. | | |
