# Supplementary material for: COVID‐19 Vaccine Effectiveness Against Medically Attended Symptomatic SARS‐CoV‐2 Infection Among Target Groups in Europe, October 2024–January 2025, VEBIS Primary Care Network
Source: Influenza Other Respir Viruses. 2025 May 21;19(5):e70120. doi: 10.1111/irv.70120 (PMC12093050; doi:10.1111/irv.70120)
Supplement: Supplementary file 2 — Table S2. Study eligibility flowchart, VEBIS primary care study, Europe, October 2024–January 2025. [file IRV-19-e70120-s001.docx]

**Table S2. Study eligibility flowchart, *VEBIS primary care study*, Europe, October 2024–January 2025**

| **Exclusion criteria** | **Patients** |
| --- | --- |
| **Patients initially recruited** | **4,777** |
| **Patients excluded** | **1,573** |
| Patients vaccinated but who received their last COVID-19 dose 1–13 days before symptom onset | 171 |
| Patients living in a residential care facility | 8 |
| Patients who were missing a swab date | 291 |
| Patients swabbed before symptom onset | 5 |
| Patients swabbed >10 days after symptom onset | 278 |
| Patients who had a missing/inconclusive SARS-CoV-2 PCR test result | 49 |
| Patients missing information on the type of test they received | 0 |
| Patients who were tested with a rapid antigen test only | 2 |
| Patients who received their last COVID-19 vaccine dose in the 6 months preceding the 2024/25 vaccination campaign | 74 |
| Patients missing information on their COVID-19 vaccination status for the 2024/25 season | 377 |
| Patients with data inconsistencies | 2 |
| Patients with a contra-indication for COVID-19 vaccination | 0 |
| Patients who received a last dose of COVID-19 vaccine during the 2024/25 vaccination campaign that was not approved by the EMA | 0 |
| Patients missing data for key covariates in the model (complete case analyses) | 4 |
| Patients recruited in countries with <10 cases or controls | 312 |
| **Patients included in the analyses** | **3,204** |
| Abbreviations: VEBIS, Vaccine Effectiveness, Burden and Impact Studies; EMA: European Medicines Agency. | |
