# Supplementary material for: COVID‐19 Vaccine Effectiveness Against Medically Attended Symptomatic SARS‐CoV‐2 Infection Among Target Groups in Europe, October 2024–January 2025, VEBIS Primary Care Network
Source: Influenza Other Respir Viruses. 2025 May 21;19(5):e70120. doi: 10.1111/irv.70120 (PMC12093050; doi:10.1111/irv.70120)
Supplement: Supplementary file 3 — Table S3. Baseline characteristics of patients included in the VEBIS primary care study, Europe, October 2024–January 2025. [file IRV-19-e70120-s004.docx]

**Table S3. Baseline characteristics of patients included in the *VEBIS primary care study*, Europe, October 2024–January 2025**

| Variables | Number of COVID-19 cases (%)   139 (4%) | Number of test-negative controls (%)   3,065 (96%) |
| --- | --- | --- |
| **Median age (IQR)** | 66 (53–73) | 63 (47–72) |
| **Age groups (years)** |  |  |
| 5–9 | 2/139 (1%) | 80/3065 (3%) |
| 10–19 | 3/139 (2%) | 121/3065 (4%) |
| 20–29 | 5/139 (4%) | 129/3065 (4%) |
| 30–39 | 8/139 (6%) | 230/3065 (8%) |
| 40–49 | 11/139 (8%) | 293/3065 (10%) |
| 50–59 | 22/139 (16%) | 435/3065 (14%) |
| 60–69 | 37/139 (27%) | 812/3065 (26%) |
| 70–79 | 36/139 (26%) | 635/3065 (21%) |
| ≥80 | 15/139 (11%) | 330/3065 (11%) |
| **Sex** |  |  |
| Female | 85/139 (61%) | 1823/3065 (59%) |
| **Presence of chronic condition (1)** | 81/134 (60%) | 2099/3019 (70%) |
| *Missing* | *5* | *46* |
| **Pregnant (2)** | 2/16 (12%) | 55/421 (13%) |
| Missing | 2 | 28 |
| **COVID-19 vaccination in the autumn/winter 2024/25 campaign** |  |  |
| Received COVID-19 vaccination as part of this campaign | 8/139 (6%) | 517/3065 (17%) |
| Did not receive COVID-19 vaccination as part of this campaign | 131/139 (94%) | 2548/3065 (83%) |
| **Vaccine received in the autumn/winter 2024/25 campaign (3)** |  |  |
| Comirnaty JN.1 | 4/6 (67%) | 283/486 (58%) |
| Comirnaty KP.2 | 0/6 (0%) | 21/486 (4%) |
| Comirnaty XBB.1.5 | 0/6 (0%) | 1/486 (<1%) |
| Comirnaty unspecified | 1/6 (17%) | 164/486 (34%) |
| Spikevax unspecified | 1/6 (17%) | 17/486 (3%) |
| *Missing* | *2* | *31* |
| **Median number of days since last COVID-19 vaccination received in the autumn/winter 2024/25 campaign (IQR) (3)** | 42 (34–47) | 41 (27–55) |
| **Lineage and sub-lineage of positive SARS-CoV-2 samples sequenced (4)** |  |  |
| BA.2.86 lineage | 29/31 (94%) | - |
| XEC sub-lineage | 19/29 (66%) |  |
| KP.3 sub-lineage | 7/29 (24%) |  |
| Other sub-lineage | 3/29 (10%) |  |
| Other lineage | 2/31 (6%) | - |
| *Missing* | *108* | - |
| **Influenza positive** | 5/139 (4%) | 470/3015 (16%) |
| *Missing* | *0* | 4 |
| **Study site** |  |  |
| France | 22/139 (16%) | 319/3065 (10%) |
| Germany | 26/139 (19%) | 181/3065 (6%) |
| Ireland | 21/139 (15%) | 577/3065 (19%) |
| The Netherlands | 16/139 (12%) | 105/3065 (3%) |
| Spain, national | 54/139 (39%) | 1883/3065 (61%) |
| **Days between onset of symptoms and swabbing** |  |  |
| 0 | 4/139 (3%) | 74/3065 (2%) |
| 1 | 28/139 (20%) | 335/3065 (11%) |
| 2 | 27/139 (19%) | 608/3065 (20%) |
| 3 | 26/139 (19%) | 627/3065 (20%) |
| 4–7 | 46/139 (33%) | 1200/3065 (39%) |
| 8–10 | 8/139 (6%) | 221/3065 (7%) |
| **Influenza vaccination in the autumn/winter 2024/25 campaign** |  |  |
| Received influenza vaccination as part of this campaign | 31/134 (23%) | 852/2982 (29%) |
| Did not receive influenza vaccination as part of this campaign | 103/134 (77%) | 2130/2982 (71%) |
| *Missing* | *5* | *83* |
| Abbreviations: VEBIS, Vaccine Effectiveness, Burden and Impact Studies; IQR: interquartile range.  (1) At least one of the following: diabetes, immunodeficiency, lung disease and heart disease.  (2) Among females of reproductive age.  (3) Among vaccinated patients.  (4) Among cases. | | |
