# Supplementary material for: COVID‐19 Vaccine Effectiveness Against Medically Attended Symptomatic SARS‐CoV‐2 Infection Among Target Groups in Europe, October 2024–January 2025, VEBIS Primary Care Network
Source: Influenza Other Respir Viruses. 2025 May 21;19(5):e70120. doi: 10.1111/irv.70120 (PMC12093050; doi:10.1111/irv.70120)
Supplement: Supplementary file 4 — Table S4a. Sensitivity analyses of pooled vaccine effectiveness against medically attended, symptomatic, PCR‐confirmed SARS‐CoV‐2 infection among target groups for vaccination, VEBIS primary care study, Europe, October 2024–January 2025. Table S4b. Sensitivity analysis: Excluding patients missing information on chronic condition, with and without adjustment. Table S4c. Sensitivity analysis: Using 7 days post‐vaccination to consider someone as vaccinated. Table S4d. Sensitivity analysis: Excluding influenza positive controls. Table S4e. Sensitivity analysis: Excluding vaccinated patients with an imputed vaccination date from TSV analyses. [file IRV-19-e70120-s002.docx]

**Table S4a. Sensitivity analyses of pooled vaccine effectiveness against medically attended, symptomatic, PCR-confirmed SARS-CoV-2 infection among target groups for vaccination, *VEBIS primary care study*, Europe, October 2024–January 2025**

**Appendix 4a. Main analysis, for comparison purposes**

| **Analysis** | **Population group** | **TSV (in days)** | **Cases** | **Median TSV (in days) among cases (IQR)** | **Controls** | **Median TSV (in days) among controls (IQR)** | **VE**  **(95% CI)** |
| --- | --- | --- | --- | --- | --- | --- | --- |
|  | Whole target group | Unvaccinated | 131 | - | 2,548 | - | - |
|  |  | Any | 8 | 42 (34–47) | 517 | 41 (27–55) | 66 (34–85) |
| *30-day TSV intervals* |  | 14–29 | 2 | 18 (17–20) | 159 | 22 (18–26) | 73 (21–94) |
|  |  | 30–59 | 5 | 43 (40–46) | 255 | 44 (36–51) | 54 (-3–83) |
| *42-day TSV intervals* |  | 14–41 | 4 | 30 (20–39) | 264 | 27 (21–33) | 70 (30–90) |
|  |  | 42–83 | 4 | 48 (45–56) | 249 | 55 (48–65) | 53 (-13–85) |
| *60-day TSV interval* |  | 14–59 | 7 | 40 (30–44) | 414 | 34 (24–46) | 64 (28–85) |
|  | Older adults (part of the age-specific target group for vaccination) | Unvaccinated | 79 | - | 1,306 | - | - |
|  |  | Any | 7 | 40 (30–48) | 448 | 41 (27–56) | 67 (33–86) |
| *30-day TSV intervals* |  | 14–29 | 2 | 18 (17–20) | 131 | 22 (18–26) | 68 (3–93) |
|  |  | 30–59 | 4 | 43 (40–47) | 225 | 44 (36–51) | 59 (0–87) |
| *42-day TSV intervals* |  | 14–41 | 4 | 30 (20–39) | 225 | 27 (21–33) | 66 (18–89) |
|  |  | 42–83 | 3 | 51 (48–62) | 219 | 55 (49–65) | 64 (4–90) |
| *60-day TSV interval* |  | 14–59 | 6 | 40 (26–44) | 356 | 35 (25–46) | 65 (25–86) |
| Abbreviations: VEBIS, Vaccine Effectiveness, Burden and Impact Studies; TSV, time since vaccination; IQR, interquartile range; VE, vaccine effectiveness; CI, confidence interval. | | | | | | | |

**Table S4b. Sensitivity analysis: Excluding patients missing information on chronic condition, with and without adjustment**

| **Analysis** | **Population group** | **TSV (in days)** | **Cases** | **Median TSV (in days) among cases (IQR)** | **Controls** | **Median TSV (in days) among controls (IQR)** | **VE**  **(95% CI)** |
| --- | --- | --- | --- | --- | --- | --- | --- |
| Excluding patients missing information on chronic condition, no adjustment | Whole target group | Unvaccinated | 126 | - | 2,506 | - | - |
|  |  | Any | 8 | 42 (34–47) | 513 | 40 (27–55) | 65 (32–84) |
| *30-day TSV intervals* |  | 14–29 | 2 | 18 (17–20) | 158 | 22 (18–26) | 72 (18–94) |
|  |  | 30–59 | 5 | 43 (40–46) | 254 | 44 (36–51) | 53 (-6–83) |
| *42-day TSV intervals* |  | 14–41 | 4 | 30 (20–39) | 263 | 27 (20–33) | 69 (28–90) |
|  |  | 42–83 | 4 | 48 (45–56) | 246 | 55 (48–65) | 52 (-15–85) |
| *60-day TSV interval* |  | 14–59 | 7 | 40 (30–44) | 412 | 34 (24–46) | 63 (26–84) |
|  | Older adults (part of the age-specific target group for vaccination) | Unvaccinated | 75 | - | 1,285 | - | - |
|  |  | Any | 7 | 40 (30–48) | 444 | 41 (27–55) | 66 (31–86) |
| *30-day TSV intervals* |  | 14–29 | 2 | 18 (17–20) | 130 | 22 (18–26) | 66 (-2–93) |
|  |  | 30–59 | 4 | 43 (40–47) | 224 | 44 (36–51) | 58 (-2–87) |
| *42-day TSV intervals* |  | 14–41 | 4 | 30 (20–39) | 224 | 27 (21–33) | 64 (15–88) |
|  |  | 42–83 | 3 | 51 (48–62) | 216 | 55 (49–65) | 64 (2–90) |
| *60-day TSV interval* |  | 14–59 | 6 | 40 (26–44) | 354 | 35 (25–46) | 64 (22–86) |
| Excluding patients missing information on chronic condition, adjustment | Whole target group | Unvaccinated | 126 | - | 2,506 | - | - |
|  |  | Any | 8 | 42 (34–47) | 513 | 40 (27–55) | 65 (32–84) |
| *30-day TSV intervals* |  | 14–29 | 2 | 18 (17–20) | 158 | 22 (18–26) | 72 (18–94) |
|  |  | 30–59 | 5 | 43 (40–46) | 254 | 44 (36–51) | 53 (-6–83) |
| *42-day TSV intervals* |  | 14–41 | 4 | 30 (20–39) | 263 | 27 (20–33) | 69 (27–90) |
|  |  | 42–83 | 4 | 48 (45–56) | 246 | 55 (48–65) | 52 (-16–85) |
| *60-day TSV interval* |  | 14–59 | 7 | 40 (30–44) | 412 | 34 (24–46) | 63 (26–84) |
|  | Older adults (part of the age-specific target group for vaccination) | Unvaccinated | 75 | - | 1,285 | - | - |
|  |  | Any | 7 | 40 (30–48) | 444 | 41 (27–55) | 66 (30–86) |
| *30-day TSV intervals* |  | 14–29 | 2 | 18 (17–20) | 130 | 22 (18–26) | 66 (-3–93) |
|  |  | 30–59 | 4 | 43 (40–47) | 224 | 44 (36–51) | 58 (-3–86) |
| *42-day TSV intervals* |  | 14–41 | 4 | 30 (20–39) | 224 | 27 (21–33) | 64 (14–88) |
|  |  | 42–83 | 3 | 51 (48–62) | 216 | 55 (49–65) | 64 (2–90) |
| *60-day TSV interval* |  | 14–59 | 6 | 40 (26–44) | 354 | 35 (25–46) | 63 (21–86) |

Abbreviations: VEBIS, Vaccine Effectiveness, Burden and Impact Studies; TSV, time since vaccination; IQR, interquartile range; VE, vaccine effectiveness; CI, confidence interval.

**Table S4c. Sensitivity analysis: Using 7 days post-vaccination to consider someone as vaccinated**

| **Analysis** | **Population group** | **TSV (in days)** | **Cases** | **Median TSV (in days) among cases (IQR)** | **Controls** | **Median TSV (in days) among controls (IQR)** | **VE**  **(95% CI)** |
| --- | --- | --- | --- | --- | --- | --- | --- |
|  | Whole target group | Unvaccinated | 162 | - | 2,794 | - | - |
|  |  | Any | 8 | 42 (34–47) | 608 | 34 (20–53) | 73 (48–88) |
| *30-day TSV intervals* |  | 14–29 | 2 | 18 (17–20) | 250 | 18 (11–24) | 85 (56–97) |
|  |  | 30–59 | 5 | 43 (40–46) | 255 | 44 (36–51) | 51 (-9–82) |
| *42-day TSV intervals* |  | 14–41 | 4 | 30 (20–39) | 355 | 23 (13–31) | 80 (52–93) |
|  |  | 42–83 | 4 | 48 (45–56) | 249 | 55 (48–65) | 50 (-20–84) |
| *60-day TSV interval* |  | 14–59 | 7 | 40 (30–44) | 505 | 30 (18–44) | 73 (46–88) |
|  | Older adults (part of the age-specific target group for vaccination) | Unvaccinated | 97 | - | 1,450 | - | - |
|  |  | Any | 7 | 40 (30–48) | 524 | 36 (21–53) | 73 (45–88) |
| *30-day TSV intervals* |  | 14–29 | 2 | 18 (17–20) | 207 | 18 (11–24) | 81 (45–96) |
|  |  | 30–59 | 4 | 43 (40–47) | 225 | 44 (36–51) | 56 (-5–86) |
| *42-day TSV intervals* |  | 14–41 | 4 | 30 (20–39) | 301 | 23 (13–32) | 76 (42–92) |
|  |  | 42–83 | 3 | 51 (48–62) | 219 | 55 (49–65) | 62 (-3–90) |
| *60-day TSV interval* |  | 14–59 | 6 | 40 (26–44) | 432 | 30 (18–44) | 72 (42–89) |

Abbreviations: VEBIS, Vaccine Effectiveness, Burden and Impact Studies; TSV, time since vaccination; IQR, interquartile range; VE, vaccine effectiveness; CI, confidence interval.

**Table S4d. Sensitivity analysis: Excluding influenza positive controls**

| **Analysis** | **Population group** | **TSV (in days)** | **Cases** | **Median TSV (in days) among cases (IQR)** | **Controls** | **Median TSV (in days) among controls (IQR)** | **VE**  **(95% CI)** |
| --- | --- | --- | --- | --- | --- | --- | --- |
| Excluding influenza-positive controls | Whole target group | Unvaccinated | 131 | - | 2,136 | - | - |
|  |  | Any | 8 | 42 (34–47) | 451 | 38 (26–53) | 67 (36–85) |
| *30-day TSV intervals* |  | 14–29 | 2 | 18 (17–20) | 153 | 22 (18–26) | 74 (23–95) |
|  |  | 30–59 | 5 | 43 (40–46) | 229 | 44 (36–51) | 56 (3–84) |
| *42-day TSV intervals* |  | 14–41 | 4 | 30 (20–39) | 245 | 26 (20–33) | 71 (31–90) |
|  |  | 42–83 | 4 | 48 (45–56) | 203 | 53 (47–62) | 56 (-7–86) |
| *60-day TSV interval* |  | 14–59 | 7 | 40 (30–44) | 382 | 33 (24–46) | 66 (31–85) |
|  | Older adults (part of the age-specific target group for vaccination) | Unvaccinated | 79 | - | 1,159 | - | - |
|  |  | Any | 7 | 40 (30–48) | 396 | 40 (26–53) | 67 (33–86) |
| *30-day TSV intervals* |  | 14–29 | 2 | 18 (17–20) | 125 | 22 (18–26) | 67 (2–93) |
|  |  | 30–59 | 4 | 43 (40–47) | 206 | 44 (36–51) | 61 (4–87) |
| *42-day TSV intervals* |  | 14–41 | 4 | 30 (20–39) | 210 | 27 (21–33) | 66 (18–89) |
|  |  | 42–83 | 3 | 51 (48–62) | 183 | 54 (48–62) | 65 (4–91) |
| *60-day TSV interval* |  | 14–59 | 6 | 40 (26–44) | 331 | 34 (24–46) | 66 (27–86) |

Abbreviations: VEBIS, Vaccine Effectiveness, Burden and Impact Studies; TSV, time since vaccination; IQR, interquartile range; VE, vaccine effectiveness; CI, confidence interval.

**Table S4e. Sensitivity analysis: Excluding vaccinated patients with an imputed vaccination date from TSV analyses**

| **Analysis** | **Population group** | **TSV (in days)** | **Cases** | **Median TSV (in days) among cases (IQR)** | **Controls** | **Median TSV (in days) among controls (IQR)** | **VE**  **(95% CI)** |
| --- | --- | --- | --- | --- | --- | --- | --- |
| Excluding vaccinated patients with an imputed vaccination date from TSV analyses | Whole target group | Unvaccinated | - | - | - | - | - |
|  |  | Any | - | - | - | - | - |
| *30-day TSV intervals* |  | 14–29 | 2 | 18 (17–20) | 159 | 22 (18–26) | 73 (21–94) |
|  |  | 30–59 | 4 | 43 (40–46) | 248 | 44 (36–51) | 60 (4–87) |
| *42-day TSV intervals* |  | 14–41 | 4 | 30 (20–39) | 259 | 27 (21–33) | 69 (27–90) |
|  |  | 42–83 | 3 | 48 (45–56) | 245 | 55 (48–65) | 62 (1–90) |
| *60-day TSV interval* |  | 14–59 | 6 | 40 (30–44) | 407 | 34 (24–46) | 68 (33–87) |
|  | Older adults (part of the age-specific target group for vaccination) | Unvaccinated | - | - | - | - | - |
|  |  | Any | - | - | - | - | - |
| *30-day TSV intervals* |  | 14–29 | 2 | 18 (17–20) | 131 | 22 (18–26) | 68 (3–93) |
|  |  | 30–59 | 4 | 43 (40–47) | 219 | 44 (36–51) | 56 (-7–86) |
| *42-day TSV intervals* |  | 14–41 | 4 | 30 (20–39) | 220 | 27 (21–33) | 64 (14–88) |
|  |  | 42–83 | 3 | 51 (48–62) | 216 | 55 (49–65) | 65 (4–91) |
| *60-day TSV interval* |  | 14–59 | 6 | 40 (26–44) | 350 | 35 (25–46) | 66 (27–86) |

Abbreviations: VEBIS, Vaccine Effectiveness, Burden and Impact Studies; TSV, time since vaccination; IQR, interquartile range; VE, vaccine effectiveness; CI, confidence interval.
